# Onyx Migration to the Middle Cerebral Artery Treated by Mechanical Thrombectomy with Subsequent Delayed Migration of Residual Onyx: A Case Report

**DOI:** 10.3400/avd.cr.26-00029

**Published:** 2026-06-24

**Authors:** Yuya Kobayashi, Yoshiki Hanaoka, Chinami Yuzawa, Minori Kurashina, Atsushi Sato, Yoshiki Sekijima

**Affiliations:** 1 Department of Neurology, Ina Central Hospital, Ina, Nagano, Japan; 2 Department of Neurosurgery, Ina Central Hospital, Ina, Nagano, Japan; 3 Department of Medicine (Neurology & Rheumatology), Shinshu University School of Medicine, Matsumoto, Nagano, Japan

**Keywords:** Onyx, mechanicalm thrombectomy, delayed migration

## Abstract

Onyx is a non-adhesive liquid embolic agent used for intracranial dural arteriovenous fistulas. We report delayed distal migration of residual Onyx after successful mechanical thrombectomy. An 81-year-old woman developed acute middle cerebral artery occlusion following Onyx embolization, which was recanalized with contact aspiration. Residual Onyx, not clearly visible on angiography, was later identified on computed tomography and progressively migrated distally. One week later, further migration caused embolic infarction in the superior division of the middle cerebral artery. Residual Onyx may be difficult to detect and can migrate because of its non-adhesive nature, requiring careful postoperative evaluation and follow-up.

## Introduction

Onyx is one of the embolic agents widely used for the endovascular treatment of intracranial dural arteriovenous fistulas (dAVFs) and cerebral arteriovenous malformations (AVMs).^1,2)^ However, technical factors related to the procedure or unexpected changes in hemodynamics may result in unintended embolization, leading to subsequent complications. We report a case in which a migrated Onyx occluding the middle cerebral artery (MCA) was successfully retrieved, achieving favorable recanalization; however, delayed distal migration of residual Onyx subsequently caused an embolic infarction.

## Case Report

An 81-year-old woman presented with a left temporal subcortical hemorrhage and extensive edema involving the left temporo-occipital region (**Fig. 1A** and **1B**). Digital Subtraction Angiography (DSA) revealed a left isolated tentorial dAVF, Borden type III (**Fig. 1C**). Transarterial embolization using Onyx was performed, achieving sufficient feeder occlusion with marked reduction of the arteriovenous shunt (**Fig. 1D**). After removal of the microcatheter used for Onyx embolization, left common carotid angiography through the guiding catheter demonstrated occlusion of the distal left M1 segment (**Fig. 2A** and **2B**). Based on the angiographic findings, embolic occlusion caused by residual Onyx within the catheter was suspected, resulting from inadequate aspiration and flushing of the guiding catheter after embolization. There was no development of the tentorial artery from the meningohypophyseal trunk and no Onyx migration into this vessel; no Onyx extension into the petrosal branch of the middle meningeal artery, which may connect to the internal carotid artery, was identified, thereby excluding these routes. The left MCA territory appeared extensively avascular, indicating impending cerebral infarction; therefore, mechanical thrombectomy using contact aspiration with a SALVA 60 aspiration catheter (Nipro, Osaka, Japan) was performed, resulting in complete recanalization with thrombolysis in cerebral infarction grade 3 (**Fig. 2C** and **2D**). Histopathological examination of the retrieved material demonstrated that it consisted solely of thrombotic components, with no evidence of Onyx, indicating that the Onyx had not been retrieved during thrombectomy.

**Fig. 1:**
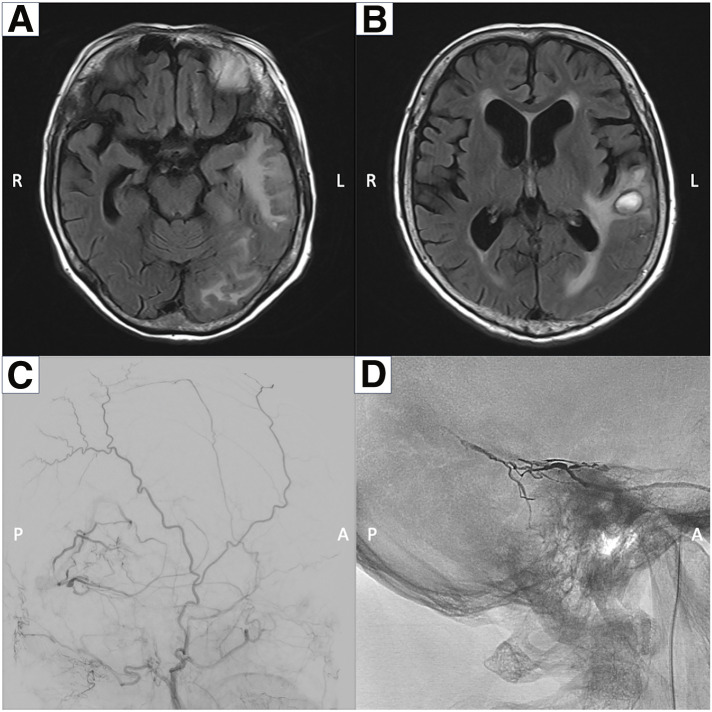
(**A**, **B**) Fluid-attenuated inversion recovery magnetic resonance imaging shows a left temporal subcortical hemorrhage with extensive edema involving the temporo-occipital region. (**C**) DSA demonstrates a left isolated tentorial dural arteriovenous fistula (Borden type III). (**D**) Onyx embolization was performed through the petrosquamous branch of the middle meningeal artery, which was the main feeder. Although reflux was observed, no migration into the petrosal branch occurred. DSA: digital subtraction angiography

**Fig. 2:**
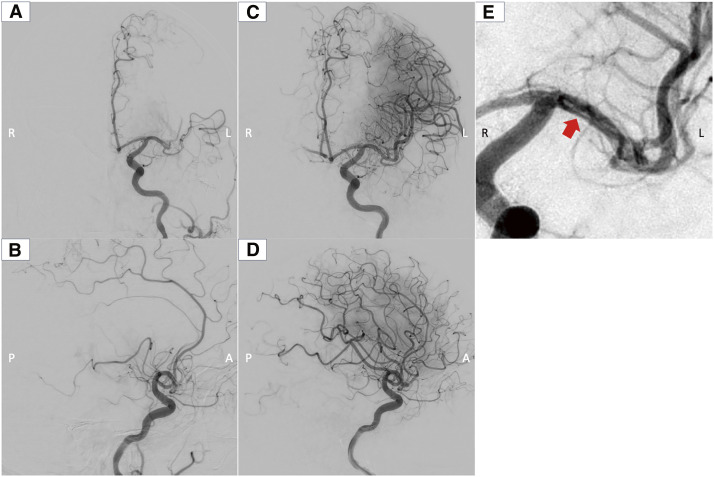
(**A**, **B**) Post-embolization left common carotid angiography after transarterial embolization with Onyx shows distal M1 occlusion of the left MCA: frontal view (**A**) and lateral view (**B**). (**C**, **D**) Angiography after mechanical thrombectomy demonstrates complete recanalization frontal view (**C**) and lateral view (**D**). (**E**) Frontal view of manual angiography from the catheter positioned in the ICA after thrombectomy, arterial phase. A retrospective detailed review of the DSA image reveals a filling defect in the MCA (red closed arrow). MCA: middle cerebral artery; ICA: internal carotid artery; DSA: digital subtraction angiography

Retrospective review of the DSA images on the following day revealed a defect at the middle cerebral artery after thrombectomy (**Fig. 2E**), which had not been recognized at the time of the procedure. Immediate postoperative computed tomography (CT) demonstrated a continuous high-attenuation structure extending from the proximal M1 segment to the superior trunk, consistent with residual Onyx (**Fig. 3A**). In contrast, magnetic resonance imaging (MRI) showed preserved vascular visualization at the corresponding site without evidence of new ischemic infarction. Considering the potential risk of complications associated with additional thrombectomy, a conservative observational approach was adopted. Aspirin was started to prevent secondary thrombosis. On CT performed the following day, the residual Onyx was found to have migrated further distally within the superior trunk (**Fig. 3B**). One week later, the patient developed worsening aphasia that had originally resulted from the hemorrhage; subsequent CT demonstrated additional distal migration of the Onyx to the peripheral portion of the superior trunk, with corresponding cerebral infarction at that site (**Fig. 3C**). Given the absence of salvageable ischemic penumbra, mechanical thrombectomy was not attempted. The patient was transferred to a rehabilitation hospital with a modified Rankin Scale score of 2.

**Fig. 3:**
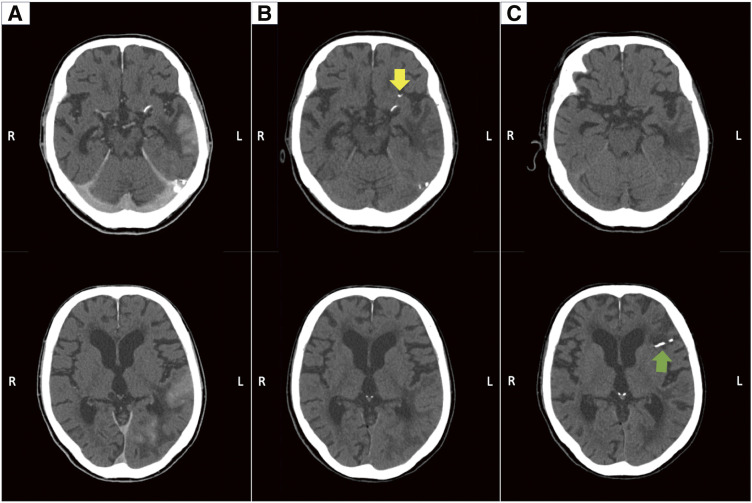
(**A**) Immediate postoperative computed tomography shows a continuous high-attenuation structure extending from the proximal M1 segment to the superior trunk of the MCA, consistent with residual Onyx. (**B**) Computed tomography performed the following day demonstrates distal migration of the high-attenuation material (yellow closed arrow). (**C**) Follow-up computed tomography obtained after worsening aphasia shows further distal migration of Onyx to the peripheral superior trunk, with corresponding cerebral infarction in the same territory (green closed arrow). MCA: middle cerebral artery

## Discussion

We treated occlusion of the left MCA caused by Onyx using a mechanical thrombectomy device and achieved favorable recanalization. However, residual Onyx subsequently migrated distally, resulting in cerebral infarction.

Two important insights can be derived from this case. First, depending on injection conditions and local hemodynamics, Onyx may form morphologies other than a cast-like structure, which can be difficult to detect on DSA. Although a retrospective, detailed review of the DSA images revealed residual Onyx, it was not detectable in the immediate postoperative period, even after careful assessment by multiple operators. Because Onyx contains micronized tantalum powder, it appears as a high-attenuation structure on CT; therefore, CT imaging was useful for evaluating residual Onyx in this case.

Second, because Onyx is a non-adhesive embolic agent, it may not be firmly attached to the vessel wall. Onyx is a non-adhesive liquid embolic agent that, after injection, initially forms a superficial solidified layer upon contact with blood and gradually solidifies from the outer surface toward the interior.^3)^ With continued injection, the remaining liquid core may disrupt the partially solidified layer, allowing further spread of the material and initiating a new phase of solidification. These properties differ fundamentally from n-butyl cyanoacrylate (NBCA), which achieves embolization through 3 principal mechanisms: (1) cast and thrombus formation, (2) adhesion to the inner vascular wall, and (3) endothelial injury.^4)^ Therefore, caution is required with Onyx, as its embolic behavior differs from that of NBCA and, because it lacks adhesive properties, delayed distal migration may occur. Reported methods for the retrieval of inadvertently migrated Onyx are contact aspiration,^5)^ stent retriever–based techniques,^6)^ and surgical removal via craniotomy.^7)^ In this case, it was unclear whether the Onyx would become entangled with a stent retriever, and there were concerns about whether lesion crossing would be feasible, as well as the possibility of fragmentation of the Onyx cast causing distal embolization. Contact aspiration was considered simpler and faster; therefore, it was selected. The optimal catheter-based retrieval strategy should be evaluated as more cases accumulate.

In the present case, residual Onyx demonstrated progressive distal migration over time, ultimately resulting in embolic complications. It is important to understand the limitations of visualizing Onyx after deployment and to recognize that its non-adhesive nature carries an inherent risk of distal migration. Whenever feasible, retrieval of unintended or residual Onyx should be considered. If residual Onyx is unavoidable, careful follow-up is warranted, with particular attention to the risk of delayed migration and subsequent embolic complications.

## Conclusion

Residual Onyx may be difficult to detect on angiography and can migrate distally, causing delayed cerebral infarction. Because of its non-adhesive properties, careful evaluation for retained material and close follow-up are essential when retrieval is not feasible.
